# Arachidonic Acid Randomizes Endothelial Cell Motion and Regulates Adhesion and Migration

**DOI:** 10.1371/journal.pone.0025196

**Published:** 2011-09-23

**Authors:** Ninna Struck Rossen, Anker Jon Hansen, Christine Selhuber-Unkel, Lene Broeng Oddershede

**Affiliations:** 1 Niels Bohr Institute, University of Copenhagen, Copenhagen, Denmark; 2 Novo Nordisk A/S, Måløv, Denmark; Swiss Federal Institute of Technology Zurich, Switzerland

## Abstract

Cell adhesion and migration are essential for the evolution, organization, and repair of living organisms. An example of a combination of these processes is the formation of new blood vessels (angiogenesis), which is mediated by a directed migration and adhesion of endothelial cells (ECs). Angiogenesis is an essential part of wound healing and a prerequisite of cancerous tumor growth. We investigated the effect of the amphiphilic compound arachidonic acid (AA) on EC adhesion and migration by combining live cell imaging with biophysical analysis methods. AA significantly influenced both EC adhesion and migration, in either a stimulating or inhibiting fashion depending on AA concentration. The temporal evolution of cell adhesion area was well described by a two-phase model. In the first phase, the spreading dynamics were independent of AA concentration. In the latter phase, the spreading dynamics increased at low AA concentrations and decreased at high AA concentrations. AA also affected EC migration; though the instantaneous speed of individual cells remained independent of AA concentration, the individual cells lost their sense of direction upon addition of AA, thus giving rise to an overall decrease in the collective motion of a confluent EC monolayer into vacant space. Addition of AA also caused ECs to become more elongated, this possibly being related to incorporation of AA in the EC membrane thus mediating a change in the viscosity of the membrane. Hence, AA is a promising non-receptor specific regulator of wound healing and angiogenesis.

## Introduction

For many physiological processes, it is of the utmost importance that the cells move in a directed fashion biasing their motility in response to the environment. One such process is angiogenesis, the formation of new blood vessels, which is mediated through the directed migration and adhesion of endothelial cells. Angiogenesis is not only an essential part of wound healing and a prerequisite for metastasis [1], [2], it is also seen in relation to pathologies such as rheumatoid arthritis, age-related macular degeneration, and pathological diabetic blindness [2], [3]. For these reasons, there has been a considerable interest in the adhesion and biased migration of cells, e.g., how these processes depend on interaction, or lack of interaction, between the cell and its environment [4], [5]. During wound healing, epithelial cells migrate collectively into vacant space in a complex fashion. Their motility is characterized by a duality between collective and individual epithelial cell behavior, and individual leader cells can be identified [6]. During the collective EC migration, considerable traction forces are exerted [7]; they arise predominantly many cell rows behind the leading front edge, so though the leader cells play an important role in cell guidance, the physical forces they exert are only a small part of that exerted by the entire migration EC monolayer [8].

Arachidonic acid (AA) is an amphiphilic compound affecting EC migration through non-receptor specific means [9], [10]. As a constitute of the phospholipids in cell membranes, AA occurs naturally within all cells, but it can also act as a signaling intermediate during inflammation [11]. This makes AA a particularly interesting target for angiogenesis regulating research.

We investigated the adhesion of individual ECs to a collagen substrate and the migration of individual ECs within a monolayer moving into vacated space, thus imitating the processes naturally occurring during inflammation and the way these processes are affected by the presence of AA. The adhesion process could be separated into two distinct phases. Both phases exhibited scaling dynamics. During the first phase, spreading progressed faster than during the second. The adhesion dynamics in the second phase were affected by the presence of AA; cell adhesion was either sped up or slowed down depending on AA concentration. Interestingly, the mean speed of individual migrating ECs within a confluent monolayer moving into vacated space was constant in time and independent of the presence of AA; however, the individual ECs lost their sense of direction. Their motion became more random, less directed, upon the addition of AA, thus affecting the extent to which the monolayer migrated into vacated space. Also, the ECs became more elongated upon the addition of AA, which might relate to their randomized motion.

## Methods

### Cell Culture

The wild-type porcine aortic endothelial cells were a gift from Steen Dissing, Department of Cellular and Molecular Medicine, University of Copenhagen, Denmark. The cells were cultivated in 6 well Multidishes (Nunclon™Δ Surface) in D-MEM∶F12 (1∶1) + GlutaMAX medium supplemented with 10% heat inactivated fetal bovine serum (FBS), 100 U/mL penicillin, and 100 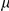
g/mL streptomycin (all from Gibco, USA). Cells from passage 5 to 15 were seeded at 25,000–100,000 cells/cm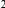, cultured in an ambient atmosphere with 5% CO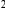 at 37

C, and grown until confluence. When cells had reached confluence, they were passaged by gentle trypsination.

### Imaging

The first stage of the cell adhesion process was imaged by confocal reflection microscopy, which works similar to RICM [12], using a TSC SP5 Leica confocal microscope. The scanning laser has a wavelength of 514 nm and an intensity at the sample of approximately 0.1 mW. The microscope was focused at the surface of the coverslip, and the depth of the confocal imaging was approximately 200 nm. The laser scanned an area of 25,000 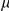
m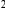 with a 42 pixel/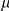
m resolution and an acquisition rate of 0.068 frames per second. This acquisition rate is relatively high and was chosen in order to have a good time resolution. The second stage of EC adhesion and individual EC migration were predominantly investigated with differential interference contrast (DIC) microscopy on a DM IRB HC Leica microscope with a frame rate of 8.6 and 0.2 frames per minute for adhesion and migration, respectively, using a Pike F-100 camera (Andor™, USA). Cell segmentation was carried out using a home-written program based on the Matlab Image Processing toolbox.

### Laser Toxicity Assay

The potential cellular damage caused by the 514 nm laser from the confocal microscope was investigated with an Automated CellTiter-Blue™ Cell Viability Assay Protocol (Promega). This assay is based on the ability of living cells to convert a redox dye (resazurin) into a fluorescent end product (resorufin) emitting at 590 nm. Viable cells retain the ability to reduce resazurin into resorufin, whereas stressed cells lose their metabolic capacity. Non-viable cells will not generate a fluorescence signal at all, hence, the intensity of the resorufin emission is indicative of a cell's metabolic health. The redox dye was fed to the cells two hours before the laser toxicity assays. The cells were then washed with serum-free medium before exposure. The illumination process proceeded exactly as described under ‘Imaging’, that is, with a relatively high scanning rate.

### Adhesion Assay

The ECs were suspended in serum-free media with the desired concentration of AA and incubated for 30 minutes at 37

C with periodic swirling before being flushed into the adhesion chambers. The adhesion chambers were perfusion chambers consisting of two coverslips separated by parafilm and vacuum grease. The lower glass slide was coated with collagen IV by spreading 10 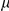
L of 1 mg/mL collagen IV (Sigma, USA) on the surface and leaving it to air dry in the culture hood. The dynamics of cell adhesion were monitored either by confocal interference reflection microscopy (514 nm) for short cell-surface contact times, or by DIC microscopy for longer cell-surface contact times of up to many hours. The combination of these methods enabled us to study cell adhesion both from the very first seconds of contact and throughout the adhesion process, which could take several hours. The outlines and areas of individual ECs were determined using a custom made MatLab routine. The adhesion assay was conducted for at least 8 individual adhering cells for each AA concentration.

### Migration Assay

EC migration was studied using razor wound assays which are commonly used as wound or angiogenesis model systems. ECs were grown on a Collagen IV substrate at 37

C until they created a confluent monolayer. Half of the cells of the confluent monolayer were then removed by gently pressing a sterile razor blade down through the endothelial monolayer and sweeping it laterally along the surface to remove cells on one side of the demarcation line. The newly vacated area was re-coated by carefully applying 1 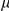
L collagen IV along the razor wound edge and left to dry for 5 min in the incubator. Then, the remaining cell monolayer was washed with, and left to migrate in, serum-free media. AA of the desired concentration was added to this serum-free media. The migration of the endothelial cell front at 37

C was monitored for 24 hours through DIC microscopy. In each migration experiment, we counted the number of cells that had migrated across the demarcation line, i.e., number of migrated cells (NMC), and we monitored the progress of the monolayer edge, as well as the progression of randomly chosen individual cells along the edge using TrackJ (from ImageJ).

### Arachidonic Acid

(A3555 from Sigma-Aldrich, USA) was added to the media. The AA was from porcine liver with a purity 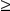
99% and suitable for cell culture. The AA was soluble in absolute ethanol, and the stock solution was diluted down to 328.44 mM before being added to the media. The media concentrations of AA used were: 0 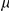
M (control), 20.53 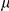
M, 41.05 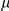
M, 61.58 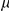
M, and 82.11 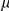
M. Within this interval of AA concentrations, the proliferation rate and general healthy appearance of EC were not affected by the presence of AA in the media (see Figure S1). For the adhesion assays, the cell density in the suspensions was kept constant for all of the experiments so that the concentrations of AA used were comparable between samples and roughly corresponded to 0 (control), 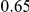, 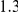, 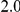, and 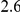 billion AA molecules per cell in the solution, respectively. The same concentrations of AA were used in the migration assays.

### Statistical methods

In both the adhesion and migration assays, a Welch's T-test [13] was used to compare the effect of two different AA concentrations. A Welch T-test is a Student's T-test in which the two groups can have different variances. We judged the differences among two compared groups to be statistically significant if p
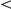
0.05.

## Analysis

### Adhesion

The cell adhesion area is the area of contact between the cell and the substrate. It evolves through several phases, with each phase exhibiting a distinct scaling behavior in the dynamics [14], [15]. Hence, the evolution of the cell area, 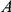, as function of time, 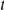, follows:
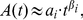
(1)where the scaling exponent, 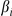, characterizes phase 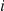, and 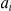 is the corresponding constant of proportionality.

In scaling analysis it is custom to introduce a time-lag, 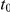, as a fitting parameter [15] because the exact initiation of the dynamical process is unknown. In our case, the physical significance of 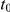 is the time elapsed between initiation of the adhesion process and initiation of the image acquisition. With 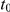 incorporated, equation 1 becomes:
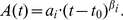
(2)


In equation 2, 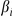 is a measure of how fast the dynamical process is progressing, the larger 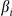, the faster the spreading of the cell's area. If 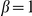 the proportionality constant 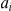 has units of m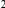/s, which are the same units as a diffusion constant. However, 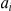 can also be interpreted as the cells' binding energy per unit area times its typical length and divided by its viscosity [15].

### Migration

If the cell performs a normal Brownian diffusion during migration its average distance traveled, 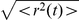, as a function of time, 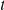, can be written:
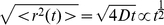
(3)where 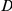 is the diffusion constant if the motion takes place in two dimensions.

If a cell follows a directed motion characterized by a velocity, 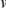, its displacement, 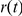, as a function of time can be described as
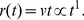
(4)


Often, equations 3 and 4 are combined into one equation which provides a simple and convenient way to describe both normal diffusion, directed motion, and all types of dynamics between those two extremes:
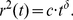
(5)


The scaling exponent, 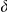 can be used to classify the dynamics: For 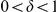 the motion is subdiffusive, for 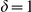 it is normal Brownian motion, for 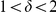 it is super diffusive, and for 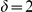 the motion is directed. 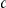 is a constant, as apparent from equations 3 and 4, 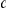 can be biologically interpreted as an effective diffusion constant (if 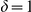) or as 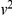 (if 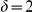).

## Results

### Adhesion

**Figure 1:**
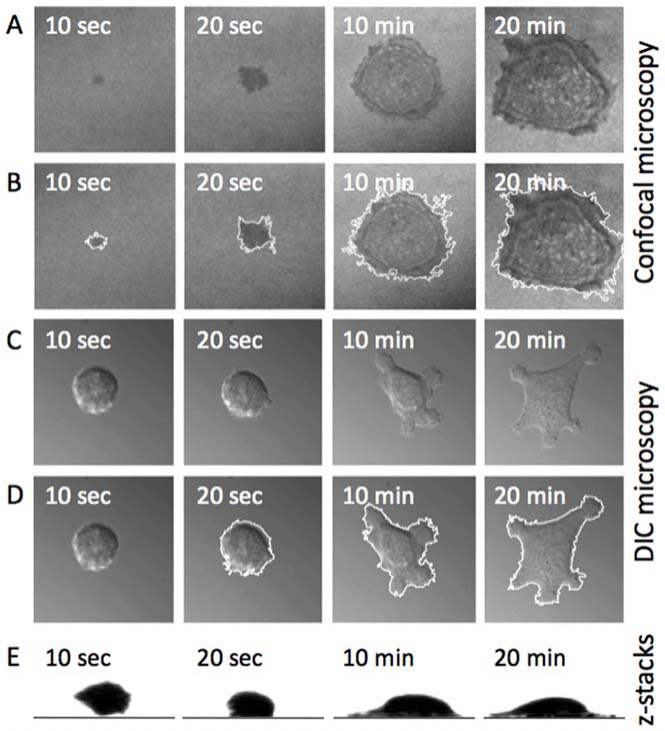
Adhesion of endothelial cells on a collagen surface. (A) Confocal reflection microscopy pictures of an adhering cell. (B) Edge detection of the cell shown in panel (A). (C) Differential interference contrast (DIC) microscopy of an adhering cell. (D) Edge detection of the adhesion area of the cell shown in panel (C). (E) Profile of an adhering cell (in a plane orthogonal to the surface).

Adhesion of individual cells to a collagen IV coated surface was studied both by DIC microscopy and confocal reflection imaging. Panels A and B in Figure 1 show confocal imaging of the adhesion process of one cell at four different times. The microscope was focused at the surface of the coverslip, and the depth of the confocal reflection imaging is approximately 200 nm. The outline of the cell's adhesion area was found through image analysis (white outlines in panel B). Panels C and D show DIC imaging of another individual EC adhering to a collagen IV coated surface. DIC imaging, however, has a significantly larger depth of the focal region than confocal reflection microscopy. Hence, DIC provides the image of the largest circumference of the cell in a plane orthogonal to the direction of the light. A z-stacked 3D confocal imaging of an adhering cell (panel E Figure 1) reveals that the ECs are still spherical when they initially make contact with the substrate, and only after a certain time does the circumference of their adhesion plane exceed the initial diameter of the suspended cell. This implies that the first stages of the adhesion processes cannot be visualized through DIC microscopy; however, they can be visualized by confocal reflection microscopy. For the latter stages of adhesion, in which the largest circumference of the cell is the adhesion area, DIC is well-suited for non-invasive visualization of the adhesion process.

Confocal microscopy could have the disadvantage of causing phototoxic effects in live cells upon extensive exposure over long timescales. Therefore, we quantified the damage of confocal visualization of adhering cells through a laser toxicity assay. As shown in Figure S2 the cells could safely be visualized by RICM using a relatively high scanning frequency (0.068 frames per second) for at least 1000 seconds. As the phototoxic effect depends on the integrated energy deposited in the cell, probably, the photo toxic effect could be minimized by choosing a lower acquisition frequency. However, in order to have a good temporal resolution we choose this relatively high acquisition frequency and as the first phase of cell adhesion (P1) lasted less than 1000 seconds it could safely be visualized through RICM without affecting the cell's metabolism.

**Figure 2:**
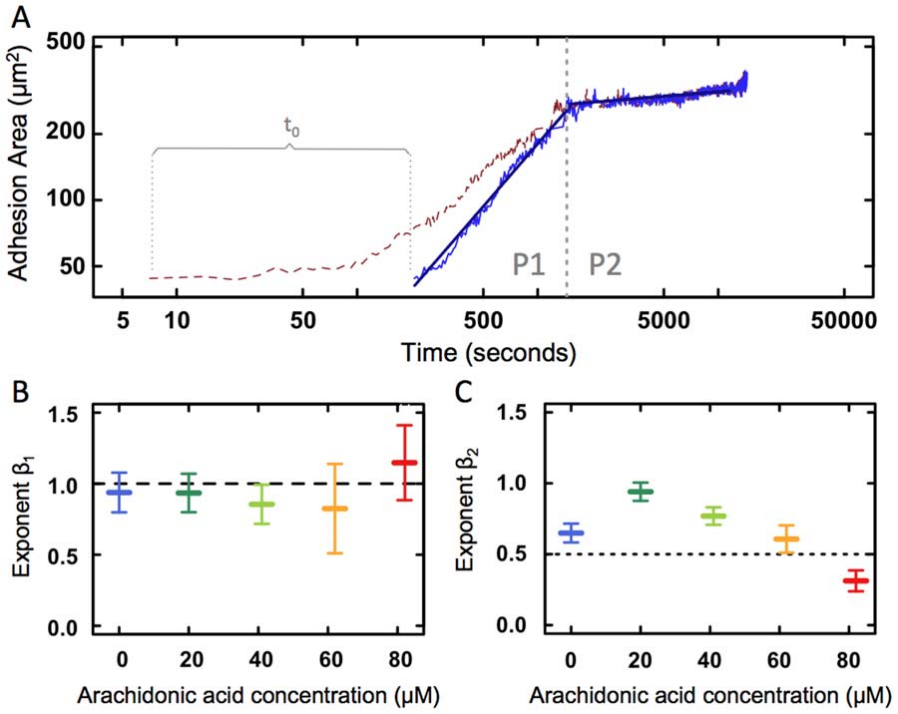
Adhesion dynamics. A) The dashed brown line shows the raw adhesion areas for an individual cell as a function of time. The full blue line shows the data after subtracting a lag-time, 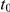, denoting the time elapsed between initiation of cell adhesion and start of the imaging process. The two dark blue straight lines show fits of equation 2 to the data in phase P1 and P2 (separated by vertical dashed grey line), respectively. B) 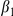 characterizing the adhering cell's spreading dynamics through P1 as a function of AA concentration. Data is acquired by confocal reflection microscopy. A dashed line is drawn at 1, the value predicted by [15]. C) 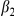 characterizing the spreading dynamics through P2 as a function of AA concentration. Data is acquired by DIC. A dotted line is drawn at 0.5, as predicted by [15]. Each datapoint shown is the mean of at least 8 independent experiments. Error bars denote one standard error of the mean (SEM).

A double logarithmic plot of the raw adhesion areas versus time appeared to have three distinct scaling regions (brown dashed line in Figure 2A). When the fitted lag-time, 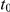, was subtracted from time 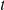 (as described by equation 2), the data showed two clearly distinct scaling regions, P1 and P2, shown with full blue line in Figure 2A. 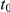 was fitted for every single cell and the average of 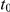 was found to be −400 seconds. The physical significance of this result is that the adhesion process typically started 6–7 minutes before the imaging was initiated. This corresponds well to the real time elapsed between flushing the cells into the chamber (first cell-surface contact) and starting the confocal reflection microscopy.

The adhesion process of ECs on a collagen IV substrate in the presence or absence of AA was characterized by two distinct phases: An initial rapidly spreading phase (P1) and a slow saturation phase (P2). Even after more than 24 hours of observation, further phases were not observed. A scaling expression (equation 2) was fitted to the cell adhesion data, thus yielding an exponent, 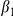, characterizing the dynamics of P1 for each adhering cell. The scaling exponent of P1, 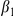, is plotted as a function of AA concentration in Figure 2B. 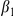 is independent of AA concentration with an average value of 0.95
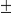
0.37. This value is indistinguishable from 1 (dashed line in Figure 2B), as predicted by [15]. Also, the proportionality constant, 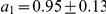, was identical for all cells and for all AA concentrations. Hence, the ratio between binding energy, typical length, and cell viscosity, appears independent of AA concentration. The phase transition between P1 and P2 occurred when the adhesion area became larger than the largest circumference of the cell. The entire second phase of cell adhesion could therefore be observed through DIC, which is essentially non-invasive. The cells observed through DIC had not previously been exposed to any laser light.

The second phase (P2) was much longer than P1 and could last for several hours until the cell was fully spread onto the substrate. As in P1, the proportionality constant, 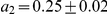, was independent of AA concentration. However, the scaling exponent, 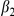, was strongly dependent on AA concentration (Figure 2C). Despite the results from the toxicity assays, we did monitor phase 2 through confocal microscopy. The values of 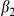 obtained by confocal imaging overlapped reasonably with those obtained from DIC imaging. At most AA concentrations, the value of 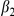 deviated significantly from 0.5 (dashed line in Figure 2C), the value predicted by the universal model put forward in Ref.[15]. The deviations of the scaling exponents, 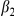, were quantified by a Welch's T-test. The resulting p-values are shown in Table 1. 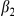 from 82 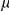
M AA was significantly lower than any other concentration on a 5 pct. significance level. Also, 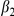 from 20 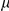
M was significantly higher than the control (0 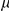
M AA), 62 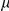
M AA, and 82 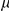
M AA. 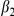 was significantly larger than 0.5 at low AA concentrations and significantly smaller at the higher AA concentrations. The effect of AA on 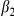 indicated that low AA concentrations sped up the spreading dynamics of the second phase (P2) in the adhesion process, whereas high AA concentrations slowed down the spreading dynamics of P2.

**Table 1:** P2 Adhesion exponents.

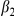 p-values:	20 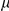 M	41 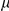 M	62 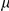 M	82 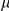 M
0 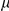 M AA	**0.0061**	0.2022	0.7237	**0.0062**
20 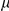 M AA	-	0.0767	**0.0158**	**0.0001**
41 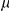 M AA	-	-	0.1858	**0.0010**
62 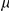 M AA	-	-	-	**0.0360**

p-values resulting from a Welch's T-test of the scaling exponents, 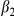, for pairs of exponents originating from different values of AA concentration. These exponents characterize the second phase (P2) of the cell adhesion process. Boldface numbers denote that the two exponents are significantly different on a 5 pct. level.

### Migration

We monitored the leading edge of an EC monolayer in a razor wound assay as it migrated out on a vacant space coated with collagen IV. The number of migrating cells (NMC) that crossed the demarcation line was counted, and the results are shown in Figure S3. The NMC was larger for 20 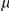
M AA than for the control, and for 62 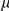
M AA and 82 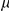
M AA the NMC was smaller than the control, in accordance with earlier observations [10]. Hence, for both adhesion and migration, 20 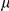
M AA has a stimulating effect and large AA concentrations have an inhibiting effect.

**Figure 3:**
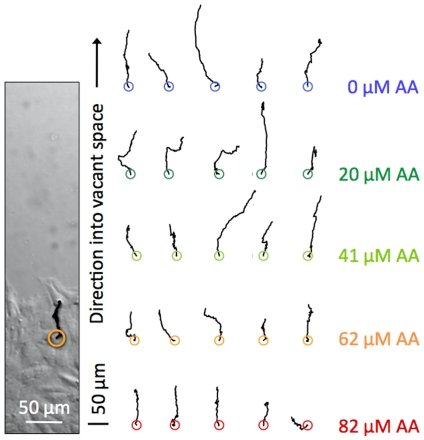
Individual EC trajectories. Left: DIC image with migration trace of an individual cell during 24 hours of migration in 62 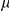
M AA media, orange circle denotes initial location. Right: Examples of trajectories of 25 individual cells with their starting points spaced out on a grid and marked with a circle. Each row shows cells from one AA concentration.

Individual cells at the edge of the monolayer were tracked during a 24 hour period. In each migration assay, cells were picked randomly among the cells along the edge of the monolayer. Examples are shown in Figure 3. The size of a typical diffusive step is too small, compared to the total length of the trajectory, to be apparent in the resolution of Figure 3, however, a zoom in on one of the trajectories at 62 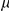
M (Figure S4) shows that the trace is not exactly persistent but appears rather random. Some of tracked and randomly chosen cells turned out to be ‘leader cells’ [6], [16], advancing into vacant space while guiding the cells behind them thereby creating expanding “fingers”. However, in some samples no distinct fingers were formed and no leader cells identified. In our analysis, the motion of the leader cells did not differ statistically from the motion of the other cells along the edge of the monolayer.

**Figure 4:**
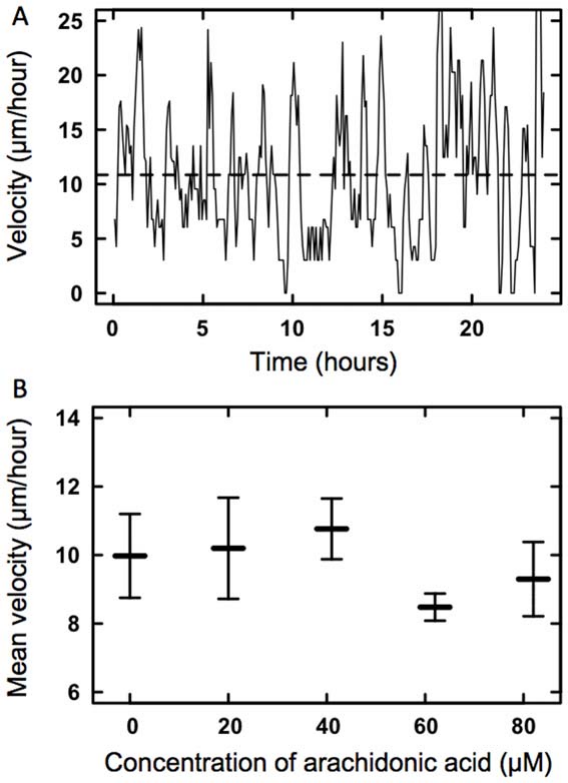
Velocity of individual ECs. (A) Instantaneous velocity of an individual EC at the edge of a monolayer migrating without any AA in the media over the course of 24 hours. (B) Mean velocity of individual ECs after 24 hours of migration as a function of AA concentration in the media. Each data point shown in B is the mean of 5 individual experiments in each of which 6–8 cells were tracked. Errorbars denote one SEM.

The velocity, 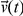, of an individual cell at time 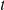 was calculated in 5 minute intervals throughout the 24 hours of observation. The instantaneous speed, 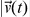, of individual cells oscillated randomly around a constant mean of 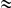
10 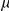
m/hour throughout the 24 hour interval (Figure 4A). There were no extended periods of stalling for any of the cells in which 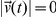. The mean speed of the individual cells at the edge of the confluent monolayer was independent of the concentration of AA (Figure 4B).

**Figure 5:**
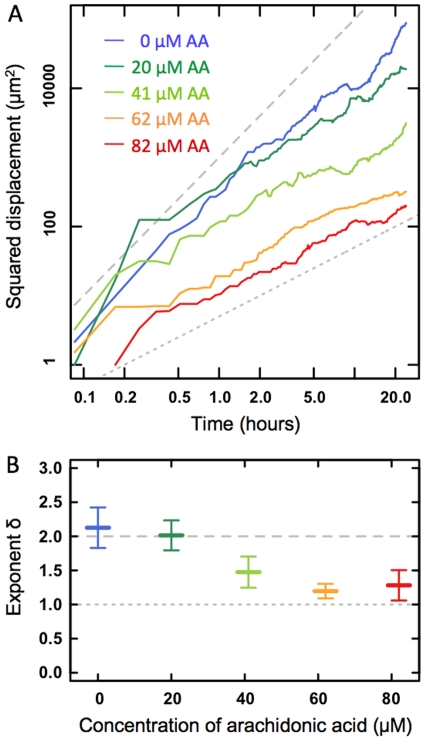
Cell displacement. A) The squared displacement (equation 5) for individual ECs at varying AA concentrations: 0 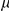
M (blue), 20 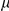
M (dark green), 41 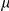
M (light green), 62 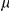
M (orange), and 82 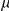
M (red). The dashed line has a slope of 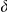 = 2 indicative of directed motion. The dotted line has a slope of 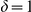 indicative of normal un-biased diffusive motion. B) The exponent, 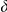, characterizes the scaling behavior of the squared displacement as a function of AA concentration. Each data point is the mean of 5 individual experiments in each of which 6–8 cells were tracked. Errorbars denote one SEM. The dashed line at 2.0 indicates the 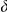 value for directed motion.

To characterize the motion of individual ECs, we calculated their squared displacement, 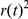 (equation 5). Examples at varying AA concentrations are shown in Figure 5A. The squared displacements obeyed the expected scaling behavior, and each individual cell's trace was fitted to equation 5, thus providing 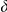 and 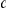 for each cell.

Though the proportionality constant, 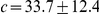, was independent of AA concentration, the scaling exponent, 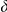, was highly dependent on AA concentration. Figure 5B shows the average values of 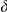 as a function of AA concentration. As visible from Figures 5 A and B, and as confirmed by a Welch's T-test (Table 2), exponents, 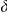, originating from assays with lower AA concentrations (0 and 20 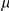
M) were significantly higher than exponents originating from assays with higher AA concentrations (62, and 82 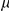
M). Hence, the ECs at the edge of the confluent monolayer had a significantly more directed motion when there was little or no AA present (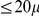
M). Large concentrations of AA (62–82 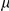
M) caused the ECs to lose their sense of direction and move in a more random fashion.

**Table 2:** Migration exponents.

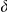 p-values:	20 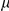 M	41 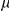 M	62 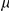 M	82 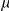 M
0 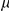 M AA	0.768	0.114	**0.024**	**0.047**
20 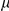 M AA	-	0.12	**0.012**	**0.04141**
41 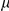 M AA	-	-	0.305	0.557
62 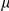 M AA	-	-	-	0.743

p-values resulting from a Welch's T-test of the scaling exponents, 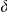, for pairs of exponents originating from different values of AA concentration. Boldface denotes that the two exponents are significantly different on a 5 pct. level.

### Morphology

**Figure 6:**
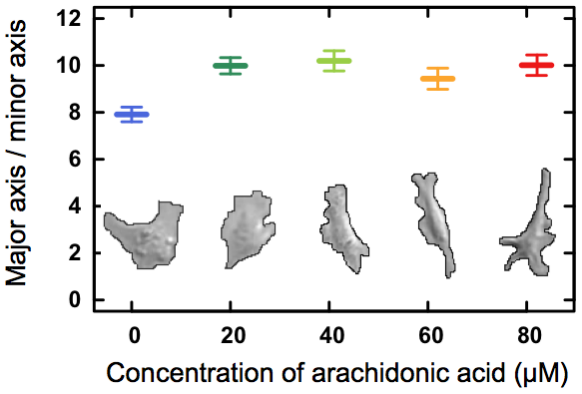
Dependence of cell shape on AA. The graph shows the elongation of individual ECs along the edge of the monolayer as a function of AA concentration. Each symbol denotes an average of at least 32 cells. Error bars denote one SEM. The pictures below show the outline of typical individual ECs at the edge of the migrating monolayer as a function of AA concentration.

The morphology of individual ECs was also affected by the presence of AA. Figure 6 shows examples of typical shapes for individual cells at the edge of the monolayer and the elongation of the individual cells' shape as a function of AA concentration. The cells at the edge of the monolayer were more elongated if they had been exposed to any of the tested AA concentrations (20–82 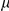
M). None of these concentration had an effect on EC proliferation rate (see Figure S1).The elongation was computed as the ratio of the major to the minor principal axis of an ellipse that had the same normalized second central moments as the shape of the individual cell. Normalizing the major axis by the minor axis or normalizing by the total area of the cell yielded identical results. A Welch's T-test showed that the cell elongation was significantly smaller for the control than if AA had been added (in any concentration). The elongations of the cells' shape for all AA concentrations were indistinguishable. Hence, addition of AA caused ECs to become more elongated.

## Discussion

Arachidonic acid (AA) is an amphiphilic compound hypothesized to affect endothelial cells through its incorporation into the cellular membrane. The resulting change in membrane composition alters the physical properties of the membrane, particularly its stiffness or microviscosity [9], [10], thus possibly affecting the functionality of some transmembrane proteins [17], [18].

The adhesion process is initiated by a contact between the EC and the substrate. As this time is random with respect to the initiation of the measurement procedure, it is reasonable to include a lag-time parameter in the scaling model (suggested by [15] and also done in [19]). Before subtracting a lag-time, the adhesion data exhibited three distinct scaling regions; however, after subtracting a lag-time, only two distinct scaling regions appeared in Figure 2A, in accordance with the universal behavior proven in Ref. [15]. Other studies, in which lag-times have not been subtracted, report three distinct scaling regions during adhesion [14], [20] or more complex dynamics [21], [22].

The first phase (P1) lasts until the adhesion area exceeds the initial circumference of the suspended cell and is sometimes denoted the passive phase. In accordance with [14], [15], [19], we found the adhesion dynamics to exhibit scaling with a scaling exponent of 1, this being independent of the presence of AA (see Figure 2B). Interestingly, the dynamics of P1 appear similar to the dynamics of an integrin reconstituted giant unilamellar vesicle adhering to a fibronectin coated surface [23], consistent with the dynamics of a membrane bound viscous shell enclosing a liquid cytoplasm [15].

In the second phase (P2), the adhesion area is larger than the initial circumference of the suspended cell, and the cell has considerably flattened. Adhesion dynamics are slower and the cells actively expend metabolic energy in order to remodel the actin filaments of the cytoskeleton into the extending lamellipodia. This phase is often referred to as the active phase. In P2, the adhesion dynamics of the control (no AA present) were consistent with a scaling exponent of 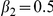 [15]. However, the presence of AA had an unpredicted and non-trivial effect on the dynamics of EC adhesion dynamics (see Figure 2C): For small AA concentrations, the scaling exponent was significantly larger than 0.5. For large AA concentrations, it was significantly smaller. Our observation of 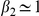 for [AA] = 20 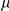
M is consistent with the exponent observed upon disruption of actin in the cortical shell of HeLa cells [15]. This is intriguing because if the predominant effect of AA is to change the viscosity of the cell membrane, then it should not affect the scaling exponent, 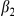, but only the constant of proportionality, 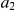 [15]. However, we observe that the presence of AA changes 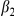 but has no effect on 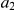.

The fact that AA can have either a stimulatory or inhibiting effect on P2 adhesion dynamics shows that its action is dependent on concentration. One possible explanation is that the cell can metabolize AA [24], and the metabolites of AA stimulate the cell. At small concentration, most of the AA is metabolized, and the stimulating effect of its metabolites can be seen. At larger concentrations, the metabolic machinery is saturated with AA and a surplus of intact AA is left to incorporate itself into the cellular membrane, changing the membrane's physical properties which might have an inhibitory effect on cell motility. A recent study [25] showed that one AA oxidation product (15-F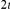-IsoP) in a purified form, and in concentrations significantly exceeding those of the present study, had a minor effect on vascular tension. None of the naturally oxidized fatty acid oxidation products had an effect on vasculation [25]. Therefore, for the present study, the potential effects of natural AA oxidation products are probably insignificant.

AA has been reported to have a regulatory effect on the number of migrating cells (NMC) at the edge of a confluent monolayer into vacated space [10]. We observed that though the presence of AA reduces the NMC, it does not affect the mean speed of the individual cells at the edge of the confluent monolayer (Figure 4). Rather, the presence of AA causes individual ECs to lose their sense of direction. The larger the AA concentration, the more random, less directed, the motion of the individual cell (Figure 5). This is also true for the leader cells [6], [16] and causes the collective migration of the EC confluent monolayer to be severely affected by AA.

The presence of AA in any of the concentrations investigated causes ECs at the edge of the monolayer to adopt a significantly more elongated shape during migration. ECs can metabolize AA into lipids that stimulate angiogenesis in various ways; by increasing the surface expression of integrins and by promoting endothelial proliferation and migration (reviewed in [26]). An increased expression of integrins could lead to a stronger EC adhesion and might explain the more elongated shape of the ECs exposed to AA (Figure 6). This elongated shape could also be a sign of an AA-induced change in actin cytoskeleton organization.

For an EC to migrate it needs to extend lamellipodia, adhere firmly to the substrate in order to relocate its center of mass, and then retract its trailing edge with an active actin based machinery. A cell that experiences difficulties in adhering to the substrate (or extending lamellipodia) will have a slower rate of adhesion in phase P2. For example, when cell adhesion is inhibited by a surface that does not sufficiently support it, the migration behavior and the phases of adhesion change completely [27]. For these types of situations, less cell spreading and adhesion goes along with a reduced stiffness of the cell [28], which is due to a reduced actin stress fiber formation. Due to the AA-induced changes of membrane microviscosity, the anchorage of adhesion receptors in the cell membrane might be influenced [17], [18], which, in turn, changes actin binding and stress fiber formation.

### Summary

The effect of arachidonic acid on the adhesion and migration of individual endothelial cells was studied using confocal reflection microscopy and differential interference contrast microscopy. The adhesion process evolved through two phases, each characterized by a scaling exponent. In the first phase, the cells spread out rapidly; their dynamics were independent of AA concentration and comparable to the passive spreading of a fluid droplet contained in a viscous shell. In the second phase, the spreading dynamics were generally slower, with low concentrations of AA having a stimulating effect and high AA concentrations having an inhibiting effect.

We analyzed the effect of AA on the dynamics and shape of individual ECs in the leading edge of a confluent monolayer moving into vacant space. The speed of an individual EC was independent of AA concentration. Without AA present, an individual cell moved in a directed fashion towards the vacant space. The presence of AA caused the individual cell to lose its sense of direction and move in a more random fashion, thus giving rise to an overall smaller number of migrating ECs into vacant space. Any of the used AA concentrations also caused the cells to become more elongated.

AA affects both EC metabolism and membrane viscosity [10]. The complex regulatory effect of AA on cell adhesion, migration, and elongation is probably caused by the metabolites of AA at small concentrations, whereas at larger AA concentrations the metabolic machinery saturates and a significant number of AA molecules incorporate into the cellular membrane, thus causing the cell membrane viscosity to decrease. The ECs adhesion process responds dynamically to applied shear stress [29], and future studies will shed light on whether the migration of ECs is similarly affected by shear stress. Further studies on AA's regulation of adhesion and migration *in vivo* will pave the way for AA to become a means to regulate angiogenesis and wound healing.

## Supporting Information

Figure S1 Proliferation rate as function of AA concentration. The EC proliferation rate appears independent of AA concentration.
(TIFF)

Figure S2 Laser toxicity assay. The red stars show the intensity of resorufin emission as a function of time during confocal reflection imaging, this is indicative of the metabolic health of the endothelial cell. The dashed red line shows the average of resorufin emission from 6 independent measurements.
(TIFF)

Figure S3Number of migrating cells (NMC) as function of AA concentration. The NMR is normalized to 100 at the value of the control (no AA present). The NMC is strongly dependent on AA concentration, e.g., at 20 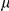
M NMR increases with respect to the control, at 60 and 80 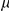
M it decreases in accordance with the observations in Ref. [10].(TIFF)

Figure S4 Zoom-in on one of the individual EC trajectories shown in Figure 3. At this spatial resolution the randomness of the trajectory is more apparent.
(TIFF)
