# Supplementary figures and images for: Arachidonic Acid Randomizes Endothelial Cell Motion and Regulates Adhesion and Migration
Source: PLoS One. 2011 Sep 23;6(9):e25196. doi: 10.1371/journal.pone.0025196 (PMC3179469; doi:10.1371/journal.pone.0025196)

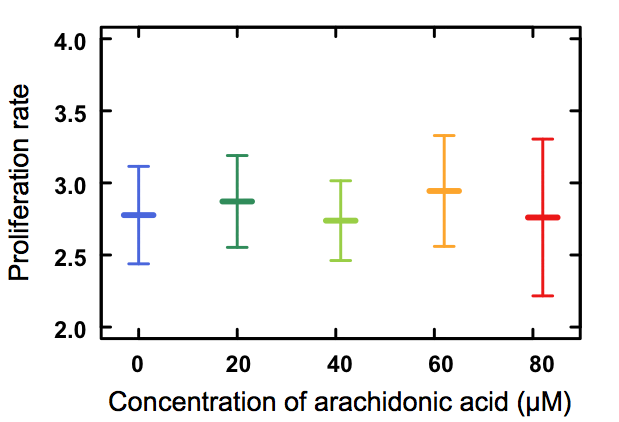

Supplement: Figure S1 — Proliferation rate as function of AA concentration. The EC proliferation rate appears independent of AA concentration. (TIFF) [file pone.0025196.s001.tif]

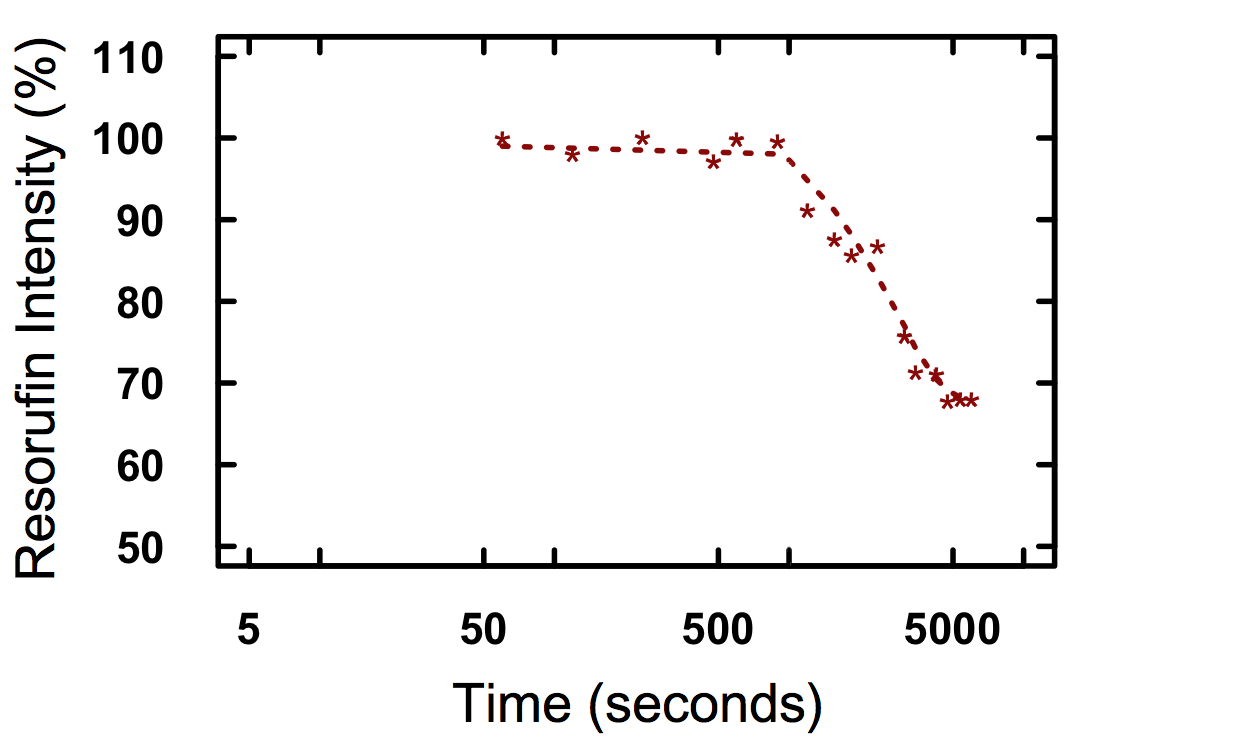

Supplement: Figure S2 — Laser toxicity assay. The red stars show the intensity of resorufin emission as a function of time during confocal reflection imaging, this is indicative of the metabolic health of the endothelial cell. The dashed red line shows the average of resorufin emission from 6 independent measurements. (TIFF) [file pone.0025196.s002.tif]

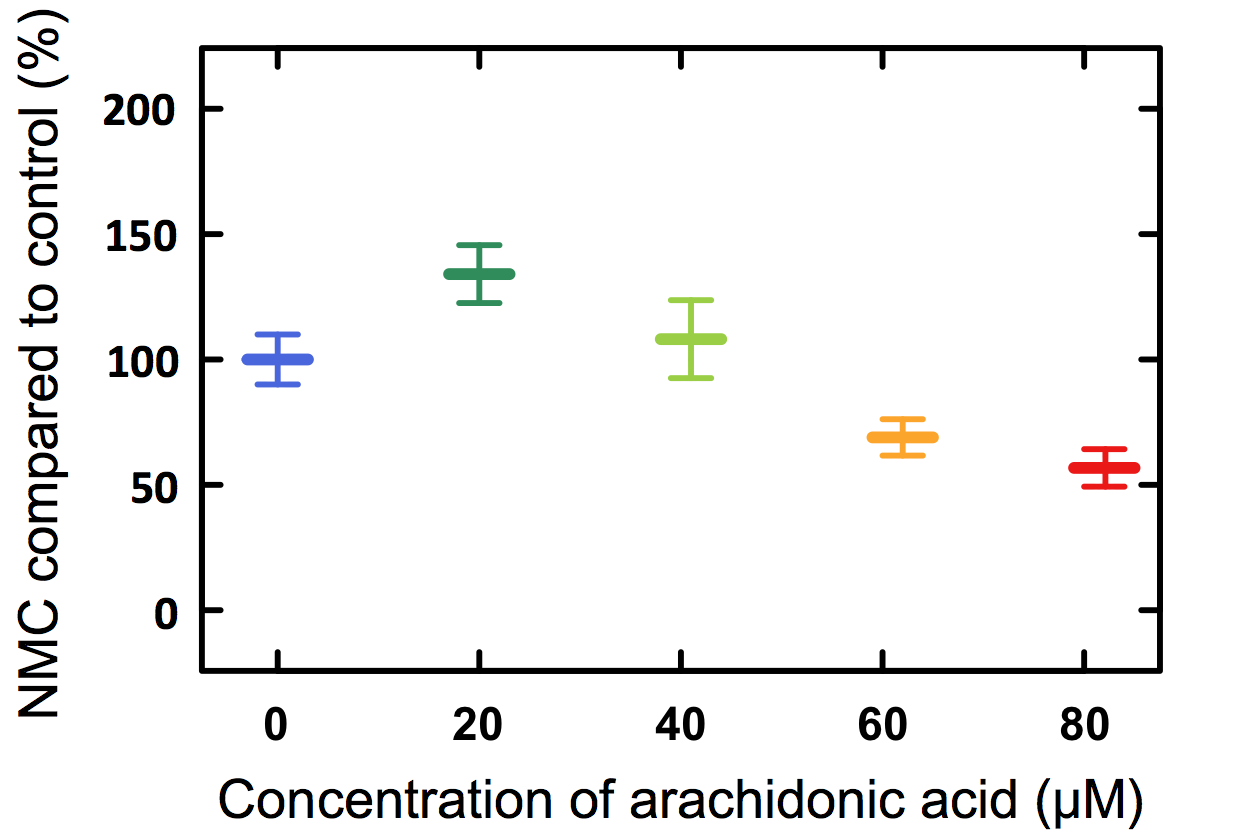

Supplement: Figure S3 — Number of migrating cells (NMC) as function of AA concentration. The NMR is normalized to 100 at the value of the control (no AA present). The NMC is strongly dependent on AA concentration, e.g., at 20 M NMR increases with respect to the control, at 60 and 80 M it decreases in accordance with the observations in Ref. [10]. (TIFF) [file pone.0025196.s003.tif]

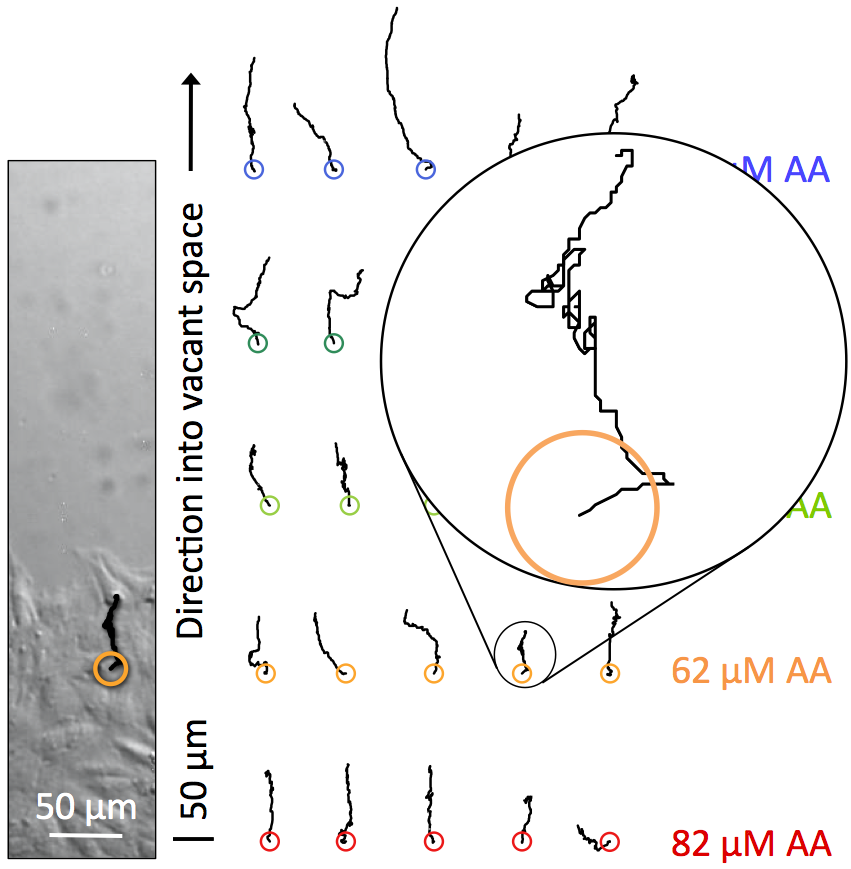

Supplement: Figure S4 — Zoom-in on one of the individual EC trajectories shown in Figure 3. At this spatial resolution the randomness of the trajectory is more apparent. (TIFF) [file pone.0025196.s004.tif]
